# The Relative Importance of Topography and RGD Ligand Density for Endothelial Cell Adhesion

**DOI:** 10.1371/journal.pone.0021869

**Published:** 2011-07-11

**Authors:** Guillaume Le Saux, Astrid Magenau, Till Böcking, Katharina Gaus, J. Justin Gooding

**Affiliations:** 1 School of Chemistry, University of New South Wales, Sydney, New South Wales, Australia; 2 Centre for Vascular Research, School of Medical Sciences, University of New South Wales, Sydney, New South Wales, Australia; Institut Curie, France

## Abstract

The morphology and function of endothelial cells depends on the physical and chemical characteristics of the extracellular environment. Here, we designed silicon surfaces on which topographical features and surface densities of the integrin binding peptide arginine-glycine-aspartic acid (RGD) could be independently controlled. We used these surfaces to investigate the relative importance of the surface chemistry of ligand presentation *versus* surface topography in endothelial cell adhesion. We compared cell adhesion, spreading and migration on surfaces with nano- to micro-scaled pyramids and average densities of 6×10^2^–6×10^11^ RGD/mm^2^. We found that fewer cells adhered onto rough than flat surfaces and that the optimal average RGD density for cell adhesion was 6×10^5^ RGD/mm^2^ on flat surfaces and substrata with nano-scaled roughness. Only on surfaces with micro-scaled pyramids did the topography hinder cell migration and a lower average RGD density was optimal for adhesion. In contrast, cell spreading was greatest on surfaces with 6×10^8^ RGD/mm^2^ irrespectively of presence of feature and their size. In summary, our data suggest that the size of pyramids predominately control the number of endothelial cells that adhere to the substratum but the average RGD density governs the degree of cell spreading and length of focal adhesion within adherent cells. The data points towards a two-step model of cell adhesion: the initial contact of cells with a substratum may be guided by the topography while the engagement of cell surface receptors is predominately controlled by the surface chemistry.

## Introduction

Cell adhesion to the extracellular matrix (ECM) plays a fundamental role in regulating cell differentiation, growth and survival [1], [2], [3] as well as cell morphology and phenotype [4]. Variations in the composition of the ECM, and organization of its components, give rise to a large diversity of topographies, each adapted to the functional requirements of a particular tissue. Like many cell types, endothelial cell adhesion and migration requires that integrin receptors on the cell surface recognize and bind to ligands in the ECM [5]. One of those ligands is the arginine-glycine-aspartic acid peptide sequence (RGD) which is present in fibronectin and other matrix proteins [6]. Engagement of endothelial cell integrins with RGD aids in a process called angiogenesis, in which quiescent endothelial cells let go of their neighbors, acquire a migratory phenotype and form new blood vessels. Because cancer growth often depends on the extent of neovascularization, understanding the fundamental properties of angiogenic transformation and the extracellular factors that contribute to this phenotype switch is important for therapeutic intervention and the design of implantable devices [7].

It is now known that the presentation of RGD ligands influences endothelial cell behavior [8], [9]. A relatively low RGD density of 6×10^6^ RGD ligands/mm^2^ (equivalent to a spacing of 440 nm) is sufficient for adhesion and spreading while higher densities of 6×10^7^ RGD ligands/mm^2^ (spacing of 140 nm) are necessary for the formation of focal adhesions and stress fibers [10]. Spatz and co-workers demonstrated that RGD spacing in the range of several tens of nanometers is necessary to promote the establishment of mature and stable integrin adhesions in fibroblasts [11], [12]. In osteoblasts, gradients in the RGD spacing induced cell polarization and migration in the direction of smaller spacings [13]. Although knowledge on the complexity of integrin-based adhesion is rapidly growing [14], these studies addressed the importance of ligand presentation on surfaces that are either flat or have ill-defined topographies.

Initially, it was thought that cell adhesion is enhanced on rougher surfaces [15] but more recent studies suggest that the influence of topography on cell behavior is more complicated. In fact, a wide range of cell responses to different surface topographies have been reported such as acceleration of cell movement, cytoskeletal reorganization and changes in gene expression [16]. Importantly, the cellular response to topography can be influenced not only by the size and density of the features but also by their regularity [17]. For random features the response appears to be cell type dependent [18]. On roughened titanium alloys, for example, epithelial cell adhesion was increased in one case [19] while others observed the opposite behavior for human osteoblasts-like cells on the same surface [21]. The type of feature also matters [20], [21], [22], [23]. Substrates presenting grooves and ridges promoted cell adhesion and migration along these features with the feature geometry dictating cell morphology [24]. Similarly, epithelial cells preferentially adhered to V-shaped grooved surfaces rather than to flat surfaces [25]. Conversely, substrates presenting ordered arrays of pillars or pits appear to impede adhesion [26], [27], [28]. Ordered topography and possibly even the directionality of features at the substrate thus seem to have an effect on the adhesion of cells.

In addition to the feature type, size, density and regularity, the choice of substrate and surface chemistry also is critical in this context [29]. Surfaces used in topographical studies have ranged from different metal alloys [30], [31], to silicon [32], ceramics [33] and polymers [34]. However, in most studies the substrates either have no adhesion ligands or lacked control over surface chemistry. Because any surface feature also introduces modifications of surface roughness and hence area, it is possible that cells adhere and migrate differently over rougher surfaces because of the alteration in presentation of surface adhesion sites. Particularly, since cells sense nano-scaled variation in RGD ligands [11], [12], it is not clear from previous studies whether the dominant effect on cell adhesion was caused by the topography or simply the greater number of adhesion sites per area. The purpose of the present work is to resolve this ambiguity.

**Figure 1:**
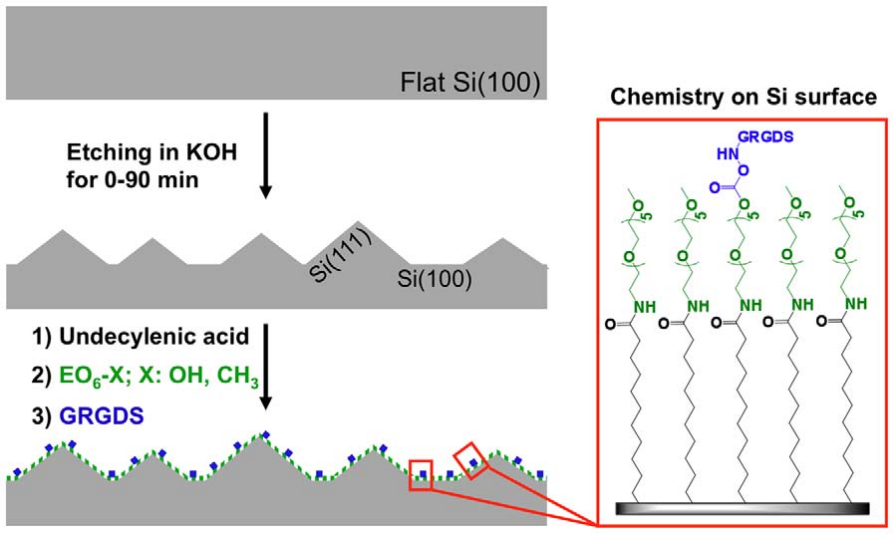
Depiction of the adhesive substratum on which surface features and chemistry could be independently controlled. Etching times (0–90 min) in KOH determine the size of pyramidal features and hence surface roughness. Subsequent functionalisation with self-assembled monolayers control RGD densities. Monolayer chemistry consists of a base layer of undecylenic acid that couples 1-amino hexa(ethylene glycol) moieties (EO_6_-X) to the surface. GRGDS peptides were grafted to activated EO_6_-OH. RGD surface densities therefore were controlled by the ratio of EO_6_-OH and EO_6_-CH_3_.

Here, we constructed a surface that allowed us to vary both the density of RGD ligands and topographical features in a highly controlled fashion. Silicon was chosen as a substrate where the density of RGD and the size and shape of topographical features could be easily and independently altered. Wet chemical etching in potassium hydroxide was employed to produce random pyramidal features whose size is controlled by the etching conditions [35]. The hydrosilylation of alkenes gives highly stable monolayers on silicon [36] to which RGD peptides were attached as integrin binding ligands (Fig. 1). The surfaces were used to investigate the role of surface topography *versus* ligand density on adhesion and migration of endothelial cells. Fluorescence microscopy images revealed that topographical features limited the number of cells that adhere onto the surfaces but cell spreading and the length of adhesion sites is only determined by the surface chemistry.

## Materials and Methods

### Wet chemical etching of silicon (100)

Silicon (100) wafers (*p* -type, orientation (100)±0.5°, 500±25 µm thick, 0.007–0.009 Ω.cm resistivity from Virginia Semi-conductors, Fredericksburg, VA USA) were cleaved into 1 cm by 1 cm pieces and rinsed in dichloromethane, ethanol and Milli-Q water and dried under a stream of argon. The samples were etched in a 20% aqueous solution of potassium hydroxide (Sigma-Aldrich, Sydney, Australia) at 50°C for 10, 30 or 90 min followed by copious rinsing with Milli-Q water.

### Preparation of the monolayers

After thorough cleaning in piranha solution (concentrated sulfuric acid/30% hydrogen peroxide = 3∶1, v/v, all Sigma-Aldrich), silicon wafers were etched in 2.5% hydrofluoric acid (HF) for 90 seconds to remove the native oxide layer. Freshly etched samples were immersed in undecylenic acid (98–100%, Sigma-Aldrich), which had previously been deoxygenated by a minimum of 5 freeze-pump-thaw cycles. The surfaces were left to react in custom made reaction vessels at 120°C for 12 h under inert atmosphere. The modified wafers were rinsed with ethanol, ethyl acetate, dichloromethane and blown dry under argon.

*Caution: piranha solution reacts violently with organic materials and should therefore be handled with extreme care.*

*Caution: HF is an extremely corrosive acid, dilute HF solutions can cause delayed serious tissue damage and should therefore handled with extreme care.*

### Coupling of the 1-amino hexa(ethylene oxide) moieties and peptide immobilization on undecylenic acid modified silicon

The surfaces were immersed for 1 hour in a 0.1 M/0.05 M aqueous solution of 1-ethyl-3-(3-dimethylaminopropyl)-carbodiimide hydrochloride and *N*-hydroxysuccinimide to convert the acid ends to succinimidyl esters and rinsed with ultra-pure water and ethanol. The samples were then incubated 12 h in 20 mM solutions in dimethylformamide containing various ratios of 1-amino hexa(ethylene oxide) (EO_6_) to 1-amino hexa(ethylene oxide) monomethyl ether (EO_6_-OMe) functional linkers which were prepared as described previously [37]. It is this crucial step in the preparation of the interfaces that gives control over ligand density. After rinsing with dichloromethane, ethyl acetate and dried under a stream of argon, the hydroxy-terminated EO_6_ molecules were activated in a 0.1 M/0.1 M solution of dry dimethylformamide of *N*,*N*′-disuccinimidyl carbonate and 4-dimethyl aminopyridine for 12 h while the methoxy-terminated EO_6_-OMe molecules remain inert to this activation step. After rinsing with dichloromethane, ethyl acetate and drying under a stream of argon, the samples were then immersed in 1 mM aqueous Gly-Arg-Gly-Asp-Ser peptide solution (Genscript, Piscataway, NJ USA) for 12 h at room temperature to form a urethane bond with the activated EO_6_ molecules. Finally, the samples were rinsed with ultra-pure water, ethanol, dried under a stream of argon and stored in dry, argon filled, sealed glass vials at 4°C. Even though the 1-amino hexa(ethylene oxide) moieties are first grafted on the surface and only EO_6_ is then modified with the RGD peptide, for convenience, the various dilution ratios will be referred to as EO_6_-RGD to EO_6_-OMe (RGD∶OMe) throughout this article.

### Cell culture

Bovine aortic endothelial cells (BAEC) were cultured in endothelial basal medium supplemented with 0.1% human epidermal growth factor, 0.1% gentamycine, 0.4% bovine brain extract and 10% fetal bovine serum at 37°C in 5% CO_2_. BAEC were transfected with green fluorescent paxillin fusion protein (eGFP-paxillin) using the lipofectamine LTX reagent (Invitrogen, Melbourne Australia) and selected in medium containing 1 mg/ml of G418 antibiotics.

### Fluorescence microscopy

Because endothelial cells are capable of secreting their own ECM proteins within 3 hours of adhesion [38], [39] and can eventually degrade it [40], adhesion times of no more than 3 hours were chosen to ensure RGD-specific interaction [41]. Therefore, BAEC were serum-starved for 18 h and re-suspended on modified silicon for 30 min or 3 h in endothelial basal medium. The adherent cells were fixed with 4% paraformaldehyde. Fixed cells were permeabilized (0.1% Triton in phosphate buffered saline, Sigma-Aldrich), blocked (0.2% fish skin gelatine, 0.5% bovine serum albumin, 0.1% saponin, in phosphate buffered saline, all Sigma-Aldrich), and stained with Alexa Fluor 555 conjugated phalloidin (Invitrogen) or with anti-vinculin (mouse, Sigma-Aldrich) followed by anti-mouse Cy3 (Jackson ImmunoResearch, West Grove, PA USA). When necessary, nuclei were stained with DAPI (Invitrogen, 0.5% in PBS, 20 min, RT). Cells were imaged on a Nikon Eclipse TE 2000-S epifluorescence microscope and on a Leica TCS SP5 confocal microscope. Quantification of focal adhesion size as well as the number of adherent cells was performed with ImageJ 1.42 image processing software with the appropriate plug-in. Z-stack images were reconstructed with Imaris imaging software (Bitplane, Zurich, Switzerland).

### Atomic force microscopy

Atomic Force Microscopy (AFM) images were obtained using a Digital Instruments Dimension 3000 AF microscope with a NanoScope IIIa controller in tapping mode at a scan rate of 0.5 Hz and Otespa silicon probe with a nominal tip radius of 10 nm (Veeco, New York USA). RMS roughness analysis of the surfaces was obtained using the roughness tool available on the Nanoscope IIIa data processing software. RMS roughness is defined as the Root Mean Squared average of height deviations taken from the mean image data plane.

## Results

Our strategy to design substrata for endothelial cell adhesion in which topographical features and the density of the integrin ligands, RGD peptides, could be controlled independently is schematically shown in Fig. 1. Etching in potassium hydroxide (KOH) generated surface topographies [35] while monolayer chemistry was employed to control RGD densities.

**Figure 2:**
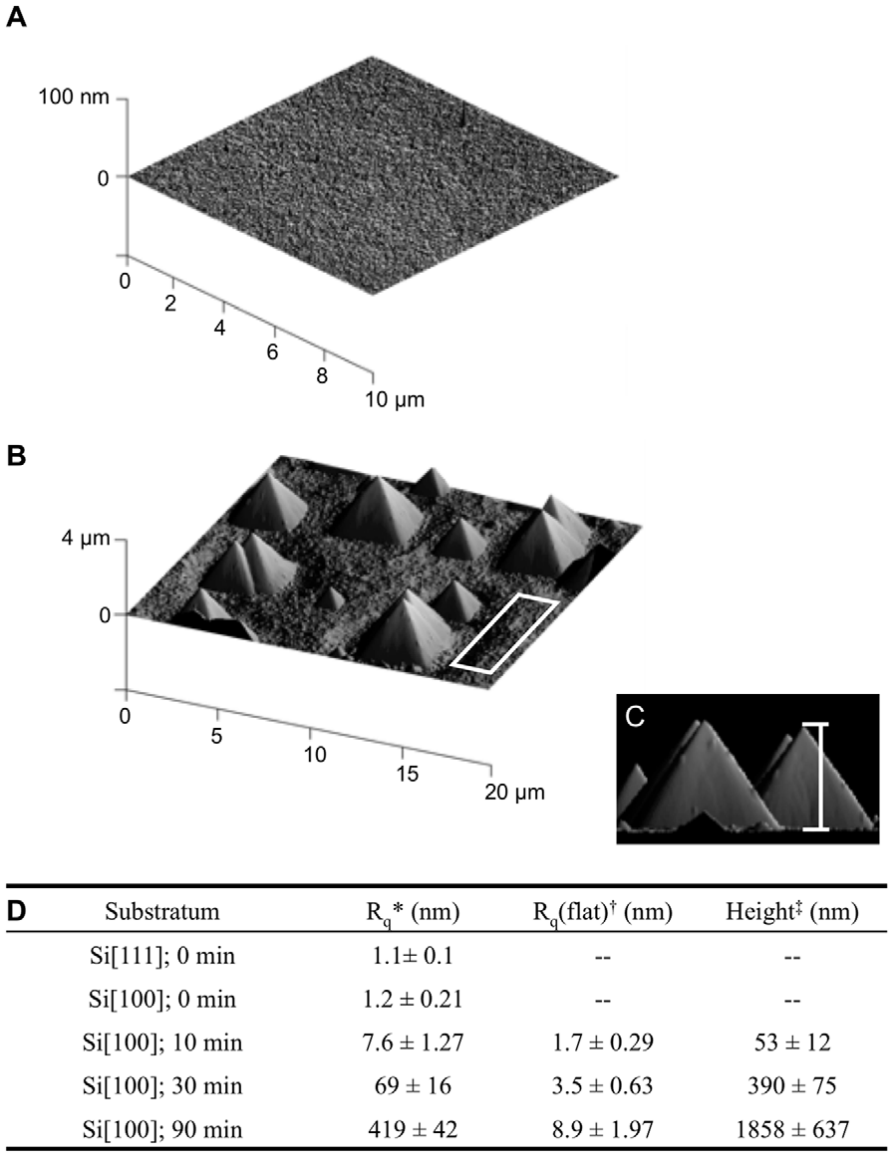
AFM images of silicon before and after etching. (A) Silicon (100) before etching and after 90 min etching in 20% KOH at 50°C (B–C). The following topographical characteristics were measured (D): *root mean square (RMS) roughness, R_q_, averaged over the entire surface; ^†^RMS roughness of flat sections of an etched surface that contains no pyramids as indicated by the white square in B; ^‡^average height of pyramids from base to apex as shown in C. For the latter, 3D surface plots were used with pitch and roll angles set to 0°, thus giving a side view of the surface. Data are means ± standard deviation of 9 independent measurements.

Prior to etching, silicon (100) surfaces are exceedingly smooth with a root mean square (RMS) roughness of 1.2±0.2 nm (Fig. 2A). Anisotropic wet etching of the silicon (100) in 20% KOH results in pyramidal features [42] with the etching time (10, 30 and 90 min) determining the feature size and RMS roughness. The pyramids can be clearly seen with atomic force microscopy (AFM) as shown in Figure 2B–C and Figure S1. The features appear as regular pentahedra composed of four lateral (111) crystallographic planes lying on the (100) base plane. This was confirmed by measuring the angle between the base plane and the lateral planes of the pyramids, which was 54.77±0.13° after 90 min of etching. This angle is in excellent agreement with the theoretical value of 54.74° between (100) and (111) crystallographic planes. The surface topography is therefore characterized by three parameters (Fig. 2D): (i) the overall RMS roughness of the surface comprising the flat parts as well as the pyramids; (ii) the RMS roughness of the flat parts only and (iii) the height of the pyramids from base to apex (Fig. 2C and Fig. S1). Etching time increased the size of the pyramids and the general roughness of the surface and also, although to a lesser extent, the roughness of the flat regions, which correspond to the (100) crystal plane of silicon. Hence, cell adhesion and migration could be compared on surfaces with nano-scaled RMS roughness (10 min of etching), intermediate roughness of tens of nanometers (30 min) and surfaces with micrometer-sized pyramids (90 min) that also had nano-scaled RMS roughness on flat regions.

**Figure 3:**
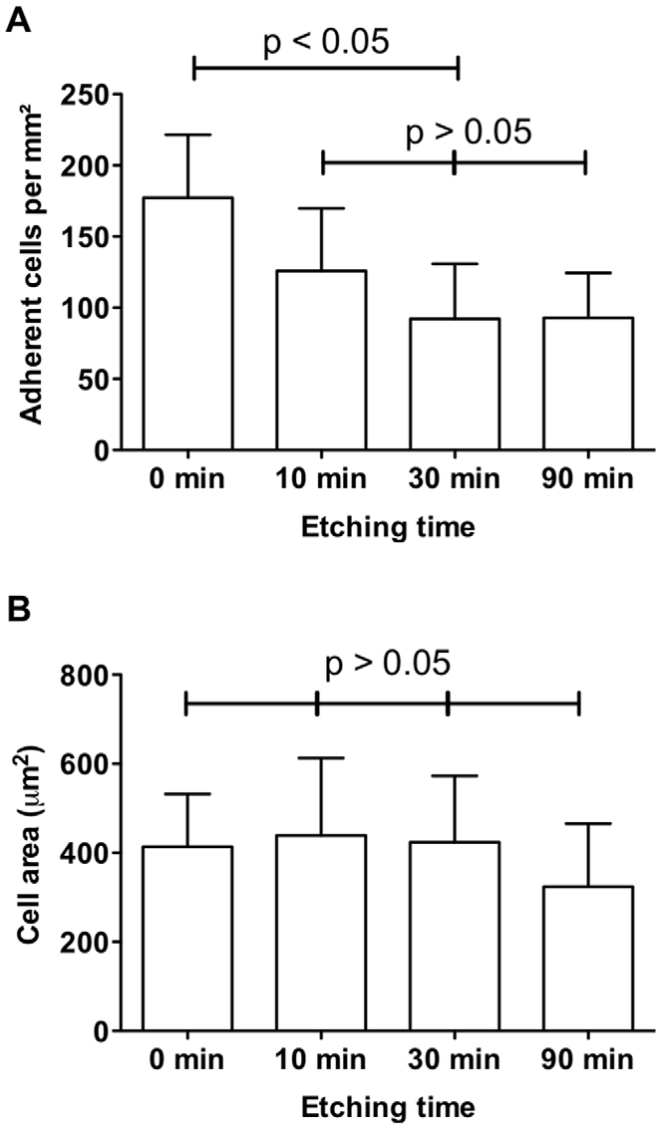
The number and cell area of endothelial cells is different on flat silicon and etched silicon. Serum-starved endothelial cells were adhered for 30 min on chemically unmodified silicon that were etched for 0–90 min. (A) The number of adherent cells per square micron is significantly different between flat (0 min) and pyramid-presenting surfaces (10–90 min) as indicated by the asterisks (p<0.05). No difference was detected on etched surfaces (p>0.05). (B) Cell area was similar on all surfaces (p>0.05). Data correspond to a minimum of three independent experiments. Error bars represent standard deviations.

The effect of surface topography on cell adhesion without adhesive ligands was investigated first (Fig. 3). Serum-starved endothelial cells were incubated for 30 min with flat silicon surfaces and silicon substrata, which had been etched in KOH for 10, 30 and 90 min. Neither substratum was modified with surface chemistry. A significant decrease in the number of adherent cells was observed for etched surfaces compared with flat, non-etched surfaces but adherence was similar on all etched surfaces (Fig. 3A). Adherent cells contained actin fibers typical for adherent cells (Fig. S2A) and spread to a similar size on all surfaces (Fig. 3B and Fig. S2B). The differences in the number of adherent cells on flat *versus* etched surfaces were the first indication that the presence of pyramids does not favor endothelial cell adhesion.

Next, the effect of RGD-modified silicon on cell adhesion was investigated. As outlined in Fig. 1, surfaces were modified by attaching a carboxylic acid terminated monolayer (undecenoic acid) to the hydrogen terminated silicon surface via hydrosilylation followed by coupling of oligo(ethylene oxide) molecules *via* formation of an amide bond [43]. The key aspect of this surface chemistry strategy is the use of two different oligo(ethylene oxide) moieties. One moiety permits further functionalisation with RGD while the other, terminating in a methoxy (OMe) moiety, does not. The surface density of RGD peptides is therefore controlled by altering the ratio of the two hexa(ethylene oxide) components is solution during the coupling reaction with undecylenic acid-modified silicon. Prior to exploring the impact of difference RGD densities and different topographies, two control experiments were performed. Firstly, the surfaces with only hexa(ethylene glycol) and no RGD ligands were incubated with cells for 30 min. These surfaces showed almost no cell adhesion and the cells that did adhere exhibited no focal adhesions (Fig. S3). This result confirmed that hexa(ethylene glycol) was effective at preventing nonspecific cell-surface interactions. The second control experiment related to the fact that both Si(100) and Si(111) crystal were revealed during the etching process. As shown in Fig. S4, the crystal orientation had no significant influence on cell adhesion.

The EO_6_-RGD to EO_6_-OMe dilution ratios were varied from 100% RGD to 1∶10^8^ RGD∶OMe (Table 1). We estimated that the increase in surface area due to the presence of the pyramids is relatively small (∼1.4-fold for the 90 min etching time). However, even small changes in surface area could have a significant impact on cell adhesion as cells sense nano-scaled variations in RGD spacing [11], [12]. Hence, we resolved the ambiguity of surface roughness on RGD presentation by expressing EO_6_-RGD to EO_6_-OMe dilution ratio in terms of RGD density per surface area. This parameter is independent of topography.

**Table 1:** Average RGD densities per surface area and RGD spacing for various RGD∶OMe ratios.

RGD∶OMe ratio	RGD	1∶10^3^	1∶10^5^	1∶10^6^	1∶10^7^	1∶10^8^	1∶10^9^
RGD density (ligands/mm^2^)	6×10^11^	6×10^8^	6×10^6^	6×10^5^	6×10^4^	6×10^3^	6×10^2^
RGD-RGD spacing (nm)	1.4	44	437	1382	4370	13820	43702

RGD stands for surfaces modified with 100% EO_6_-RGD. To calculate RGD densities, surface area per alkyl chain in the base monolayer of 0.255 nm^2^ and an overall yield of 15% for the bio-functionalisation of silicon with RGD was used. For 100% EO_6_-RGD this yields one RGD molecule per 1.7 nm^2^ surface area. For surfaces with varying ratios of EO_6_-RGD to EO_6_-OMe (RGD∶OMe ratio), the RGD density is calculated by taking the dilution ratio into account. For calculation of the RGD–to-RGD spacing, it was assumed that the RGD molecules are arranged in a hexagonal pattern.

**Figure 4:**
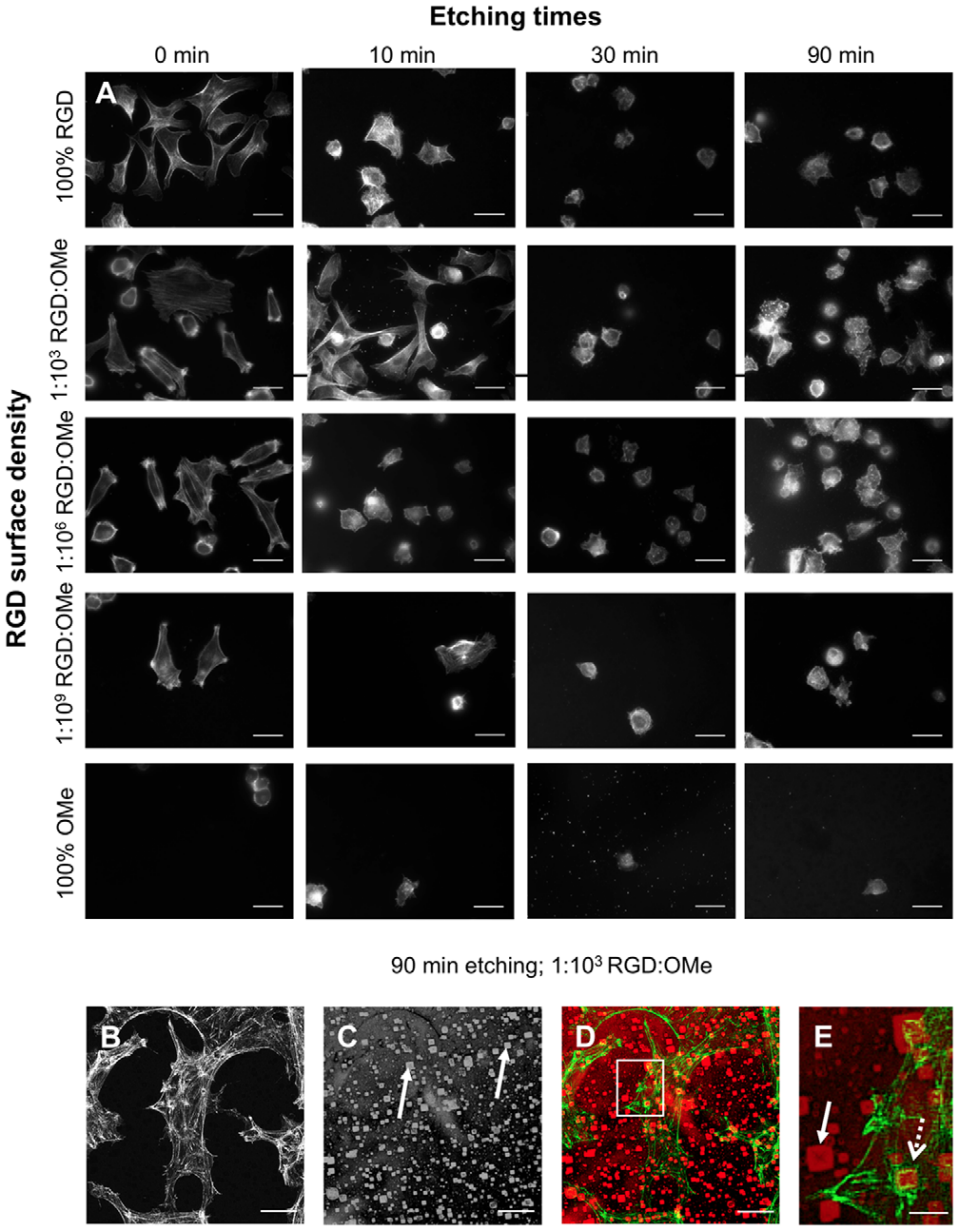
F-actin staining shows that endothelial cells adhere to flat and to etched surfaces. Serum-starved endothelial cells were adhered for 30 min on silicon surfaces with various topographies and RGD densities. Characteristics of topographical features as a function of etching times are shown in Fig. 2. RGD densities determined by the RGD∶OMe ratio are listed in Table 1. Cells were stained with phalloidin-Alexa Fluor 555 to visualize F-actin and imaged with an epifluorescence (A) and a confocal (B–E) microscope. (B–E) To view cells and the underlying substratum, images were acquired in fluorescence (B) and in bright field mode (C) at the same focal depth and merged (D–E). Zoomed region indicated in D is shown in E. In D–E, F-actin is pseudo-colored green and substrate red. Closed arrows point to the silicon pyramids; dashed arrows to agglomerates of actin. Scale bars are 20 µm (A to D) and 5 µm (H).

**Figure 5:**
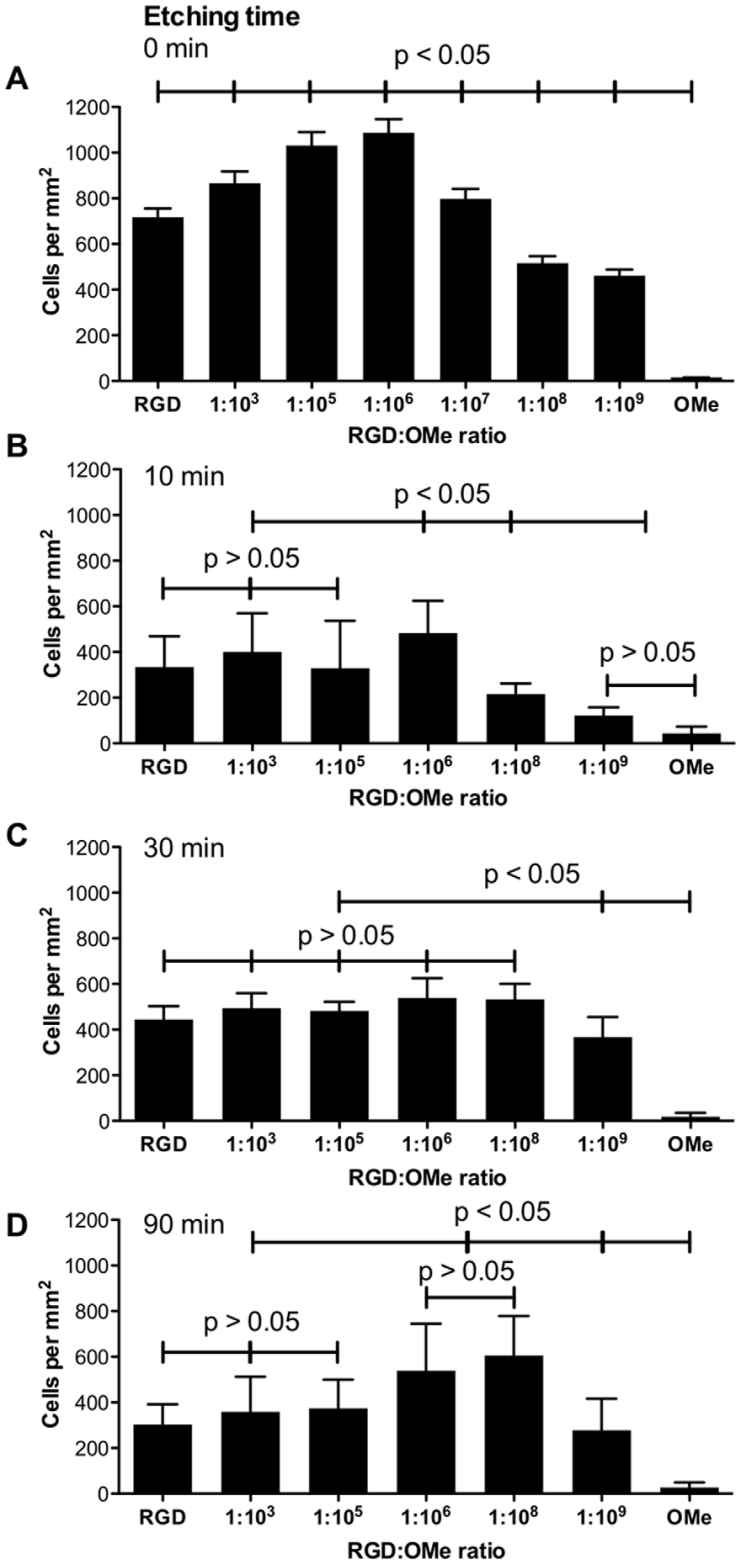
Quantification of the number of adherent endothelial cells on flat and on etched silicon surfaces. (A) Number of endothelial cells after 30 min incubation with flat surfaces (A) and surfaces etched for 10 min (B), 30 min (C) and 90 min (D) for different RGD∶OMe ratios. The corresponding RGD densities are listed in Table 1. RGD stands for 100% RGD modification or 6×10^11^ RGD/mm^2^. OMe is the anti-fouling modification and contains no RGD. Data correspond to at least three independent surface preparations and >10 images per surface. Error bars represent standard deviations. Significant differences between data points in A–D are indicated (p<0.05; p>0.05).

To quantify cell adhesion, serum-starved endothelial cells were adhered for 30 min on surfaces with various topographies and range of RGD densities (Fig. 4A). It becomes immediately apparent that both the topography as well as the RGD density determines cell number and spreading. The number of cells per square micron was counted (Fig. 5A–D). As seen on bare silicon, the presence of pyramids on RGD-modified surfaces induced a drastic decrease in the number of adherent cells compared to the non-etched flat surfaces. This decrease in cell number due to the pyramids was observed for every surface that contained RGD. For example, the number of cells on etched surfaces that were modified with 100% RGD, which is equivalent to an RGD surface density of 6×10^11^ RGD/mm^2^, had 2.1–2.6-fold fewer cells than the corresponding flat surfaces. Hence, our data clearly demonstrates that the presence of pyramids decreases cell adhesion [30], [44]. It is also noteworthy that the number of adherent cells on unmodified silicon is consistently lower than that of RGD-modified silicon (Fig. 3 and Fig. 5) as expected for specific cell-surface interactions facilitated by RGD [41]. Similarly, surfaces that were modified with anti-fouling chemistry but contained no RGD (OMe in Fig. 5) prevented cell adhesion regardless of the surface topography.

When cell numbers on surfaces with a given topography, but variable RGD density, were compared, a biphasic trend was observed. On flat surfaces, cell adhesion increased significantly until RGD densities were diluted to 1∶10^6^ RGD∶OMe or 6×10^5^ RGD/mm^2^ but any further dilution resulted in a significant decrease in cell number per square millimeter (Fig. 5A). It may be surprising that surfaces with high densities of 100% RGD and 1∶10^3^ RGD∶OMe did not enhance cell adhesion over surfaces with 1∶10^6^ RGD∶OMe. At high surface density surface crowding needs to be taken into account. A close packing of RGD peptides may provide insufficient space for efficient engagement between the ligands and the integrins in the cell membrane. The biphasic behavior was mirrored on surfaces that were etched for 10 min, where a less pronounced but statistically significant maximum of adherent cells was observed at the same RGD density as above (Fig. 5B). Hence on these surfaces, the data suggest that there is an optimal RGD density for cell adhesion. Our optimal RGD densities on flat surfaces are an order of magnitude lower than those reported previously by Massia *et al.* who found that on a RGD-modified glass substrate a density of 6×10^6^ RGD ligands/mm^2^ was sufficient for fibroblast adhesion and spreading [10].

When surfaces were etched for 30 min and therefore had an overall RMS roughness of 69±12 nm, a different behavior was observed. On these surfaces, the number of adherent cells was independent of RGD densities with the exception of very low RGD densities (Fig. 5C). This observation indicates that on surfaces with intermediate roughness, the topography is the overriding parameter for cell adhesion. While the surface chemistry is still required to provide specific anchorage points, cell adhesion was similar over a wide range of RGD densities from 6×10^3^–6×10^8^ RGD ligands/mm^2^. This behavior contrasted with cell adhesion onto surfaces that were etched for 90 min (Fig. 5D). Here, again a biphasic behavior was observed with the peak adhesion occurring at 6×10^3^–6×10^5^ RGD ligands/mm^2^. A shift to lower RGD densities for optimal cell adhesion on surfaces with large pyramids, as compared to flat surface or surfaces with nano-scaled overall RMS roughness of 7.6±1.3 nm, suggests that on these surfaces, the 3-dimensional ligand presentation facilitates optimal cell adhesion at lower ligand densities. In fact this topographical effect appears to re-introduce an optimal RGD density that was not observed on surfaces with intermediate roughness. In this context, it is noteworthy that roughness of the flat parts of the surfaces that were etched for 90 min is similar to the overall roughness of those etched for 10 min (Fig. 2D).

To investigate whether cell adhesion onto surfaces that were etched for 90 min, was dominated by the flat regions that contained no pyramids, we took fluorescent images of the F-actin organization at higher magnification (Fig. 4B–E). While adherent cells on flat and nano-scaled rough surfaces had well-defined filopodia, and had easily identifiable actin stress fibers (Fig. 4A), cells on surfaces etched for 90 min also had agglomerates of F-actin. By overlaying fluorescent (Fig. 4B, green in Fig. 4D–E) and bright field (Fig. 4C, red in Fig. 4D–E) images, we noticed that some agglomerates of F-actin were organized around pyramids (Fig. 4D). In a zoomed region (Fig. 4E), the pyramids were clearly apparent (closed arrow) with F-actin structures surrounding a pyramid (open arrow). Hence, cells did not ‘avoid’ the pyramids but adhered over both flat regions and areas that contain pyramids.

**Figure 6:**
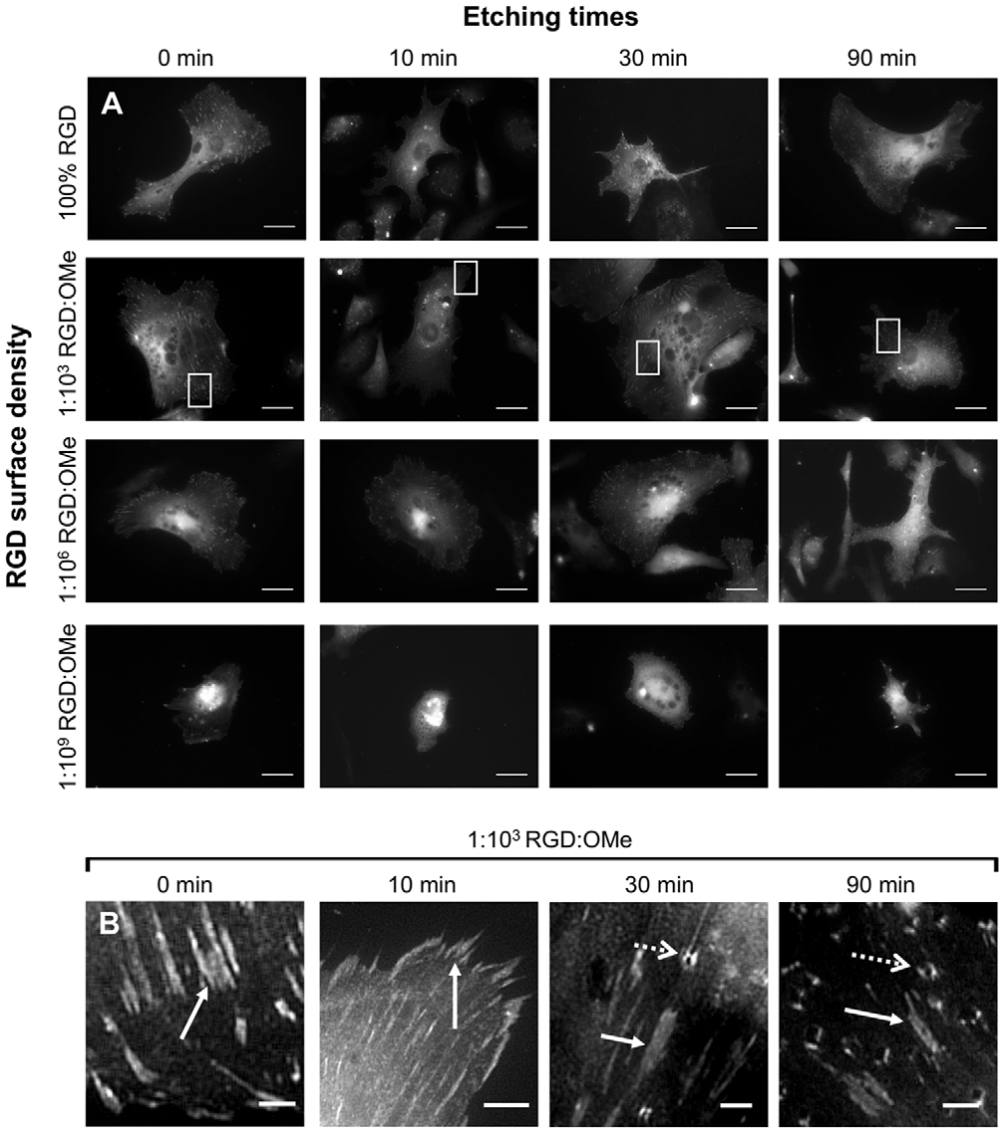
Confocal images of GFP-paxillin in adherent endothelial cells after 3 h incubation with surfaces with different topographical features, determined by the etching times, and RGD densities, defined by the RGD∶OMe ratio. Dashed arrows point to the paxillin agglomerates, closed arrows point to ‘classical’ dash-shaped focal adhesions. Scale bar 20 µm (A), 2 µm (B).

To more closely examine whether the surface chemistry affected the organization within an adherent cell, we imaged focal adhesions in paxillin-GFP transfected cells that were allowed to adhere for 3 h. Focal adhesions had a typical dash-like shape (closed arrows in Fig. 6B). At low concentrations of RGD ligands (1∶10^6^–1∶10^9^ RGD∶OMe), paxillin-GFP was mainly located in perinuclear regions, suggesting that cells on these surfaces had poorly spread. On surfaces with large pyramids and high RGD densities (100% RGD and 1∶10^3^), in addition to focal adhesions, another type of square shaped feature was observed (open dashed arrows in Fig. 6B). Although some of these features were present on the 30 min-etched substrates, they were mostly observed on the surfaces etched for 90 min. In the same way that F-actin was organized around the pyramids in some instances (Fig. 4B–E), the unusual paxillin features observed in Fig. 6 co-localized with the pyramids. The presence of both actin and paxillin on the pyramids suggests that these features are indeed focal adhesions because both proteins are part of the integrin adhesome [14].

**Figure 7:**
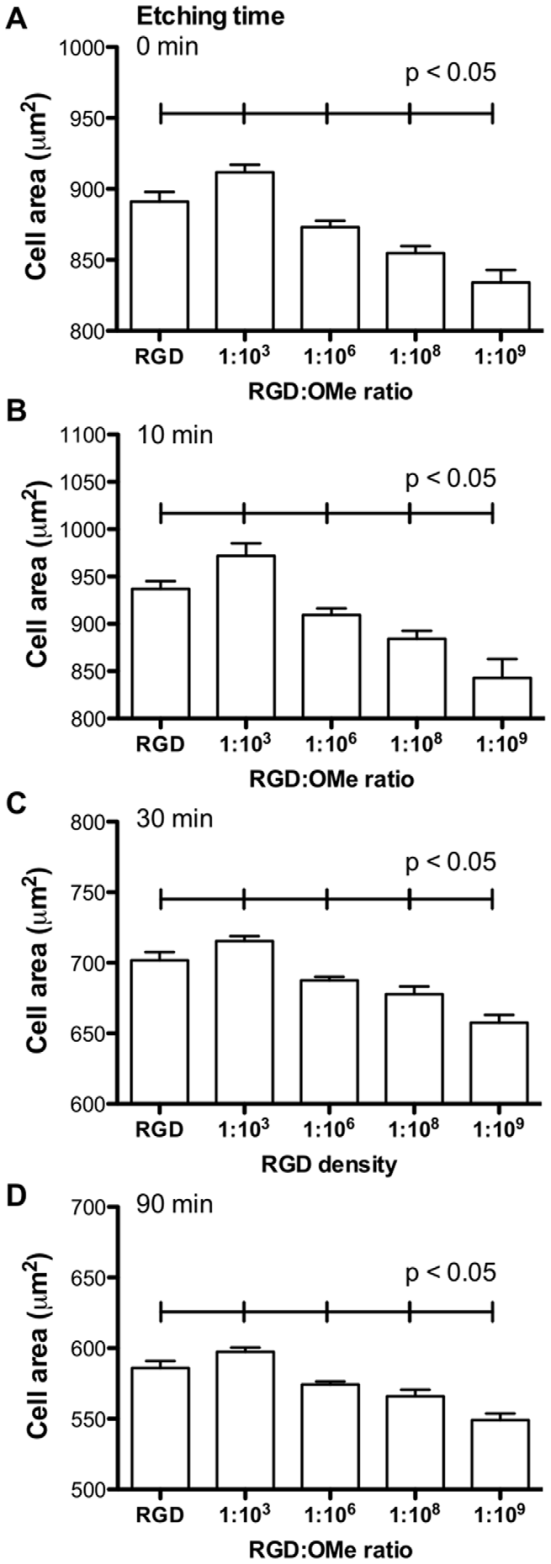
Quantification of endothelial cell area on flat and etched silicon surfaces. (A) Cell spreading of adherent endothelial cells after 3 h incubation with flat surfaces (A) and surfaces etched for 10 min (B), 30 min (C) and 90 min (D) for different RGD∶OMe ratios. In A–D, each data point is significantly different from all other data points. Data are derived from at least three independent surface preparations and >10 images per surface. Error bars represent standard deviations.

To quantify cell organization of adherent cells, we measured cell area (Fig. 7A–D). In contrast to cell adhesion, cell spreading was not affected by nano-scaled roughness, as the sizes of cells on flat surfaces and on surfaces that were etched for 10 min were similar when comparing identical RGD densities. In contrast, on 30 min- and 90 min-etched surfaces, cells spread to a lesser extent than on smoother surfaces and this correlates well with focal adhesion length, which follows the same trend (Fig. S5). The most noticeable feature of the data in Figure 7 is that the relative cell spreading is a function of RGD densities, irrespectively of surface topography. On flat and rough surface, cells spread the most on surfaces with 1∶10^3^ RGD∶OMe or 6×10^8^ RGD/mm^2^. This density was higher than the optimal density for cell adhesion as previously observed [10]. Similarly, cells had the longest focal adhesion on surfaces with 6×10^8^ RGD/mm^2^ and again, the peak was observed at the identical RGD density, independently of surface roughness (Fig. S5). Hence, we conclude that while topography critically influences the number of endothelial cells that adhere to a substratum, the surface chemistry dictates the organization within adherent cells.

**Figure 8:**
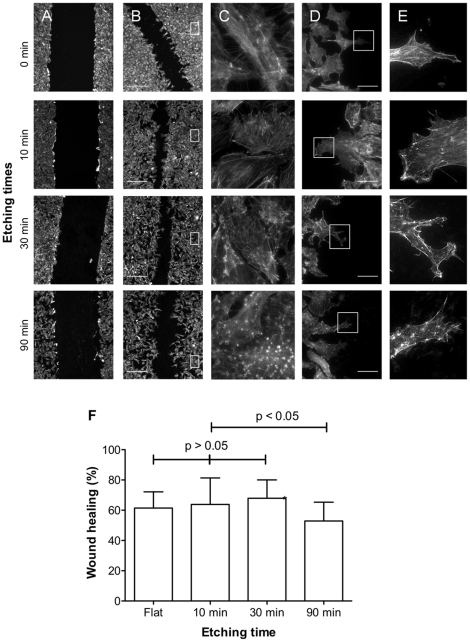
Endothelial cell migration over surfaces with different topographies, as defined by the etching time, and RGD surface densities of 6×10^5^ RGD/mm^2^ (1∶10^6^ RGD∶OMe ratio). (A) Wound immediately after scratching; (B–E) wound after a further 3 h incubation. (C) Zoomed images of cells some distance from the wound edge as indicated in B. (C–E) Zoomed of images of cells migrating into the wound. Cells were stained with phalloidin-Alexa Fluor 555. Scale bar 100 µm (A, B) and 20 µm (D). (E) Quantification of wound closer after 3 h incubation.

Finally, cell migration on flat surfaces *versus* etched surfaces was examined for a selected RGD density of 1∶10^6^ RGD∶OMe or 6×10^5^ RGD/mm^2^. Serum-starved BAEC were plated on the silicon surfaces in a confluent monolayer of cells. A ‘wound’ was created by scraping cells off the surface with a plastic pipette tip, washed to remove loose cells and then placed in fresh medium for up to three hours (Fig. 8A). Scratching with a plastic pipette tip did not remove the surface chemistry. The initial width of the wound was 317±21 µm, which is consistent with the width of the pipette tip of 300 µm. Because of the smooth edges of the wounds, changes in width and regularity can easily be detected. After 3 hours of migration (Fig. 8B), the wound widths were visibly reduced. The extent of healing was not uniform across all surface topographies with less healing on the surfaces etched for 90 min than on surfaces with shorter etching times. Stationary cells away from the edge of the wound (Fig. 8C) were not orientated in any particular direction but revealed differences in F-actin structure depending on topography as discussed above. Cells at the edge of the wound (Fig. 8D–E) were elongated in the direction perpendicular to the wound on all surfaces, suggesting that wound closure is due to the migration of cells and not mitosis. Although to a lesser fashion than observed for stationary cells, agglomerates of actin were visible on migrating cells on surfaces etched for 90 min, suggesting that cells may use pyramids for migration.

Quantification of the extent of wound closure (Fig. 8F) showed that cell migration speed on flat surfaces and surfaces with low and intermediate roughness (10 min and 30 min etching) is similar. This is not the case on 90 min-etched surfaces with the identical RGD density where cells had migrated significantly more slowly. This indicates that while topographical pyramids in the range of tens and hundreds of nanometers in size had no effect on cell migration, micrometer-sized pyramids hinder cell adhesion and migration. It is therefore possible that large features constitute a physical barrier for endothelial cells. However, when features are sufficiently small as to not function as a barrier, migration speed is independent of roughness and is likely to be governed by the subcellular organization, which in turn is predominately influenced by the RGD surface density.

## Discussion

This study sought to ascertain the relative importance of topography and surface chemistry on the adhesion of cells to solid substrates. This was achieved by etching silicon substrates to give well-defined topographies exhibiting pyramidal features which are a mixture of Si(100) and Si(111) crystal faces, but have no particular topographical orientation. These surfaces could subsequently be modified with highly stable monolayer chemistry via hydrosilylation of alkenes. The surface chemistry was terminated with hexa(ethylene oxide layers) (EO_6_) that resist cell adherence and terminated either in OMe or the integrin binding ligand, RGD. By varying the topography and the ratio of RGD ligands∶OMe, surfaces could be compared with the same RGD density per surface area but different topographies or *vice versa*, surfaces with different topographies and the same density of RGD ligands per surface area. Control experiments showed that the surface architecture enabled specific RGD-cell interactions and that crystal orientation had no influence on the number of adherent cells. We found that firstly, fewer endothelial cells adhered onto roughened surfaces compared with molecularly smooth surfaces but in the range between nanoscale and microscale roughness, the degree of overall surface roughness did not influence the number of adherent cells. Secondly, on flat and surfaces with nano-scaled roughness, we measured an optimal RGD density for cell adhesion of 6×10^5^ RGD/mm^2^. Only on surfaces with micro-scaled pyramids was the presentation of ligands such that an RGD density below 6×10^5^ RGD/mm^2^ was optimal for adhesion. Thirdly, cell spreading and focal adhesion length was greatest on surfaces with 6×10^6^ RGD/mm^2^ irrespectively of presence of pyramids and their size. And finally, only surfaces with micro-scaled pyramids hinder cell migration.

While the effect of RGD density on cell adhesion is relatively well characterized [11], [12], [14], the effect of topography is more difficult to delineate. Rougher surfaces expose more ligands per geometric area so that an adherent cell of identical size is exposed to a higher number of ligands on rough surface than on a flat surface. Indeed, initially it was thought that surface roughness enhances cell adhesion [15]. However, a more complex picture has emerged [16] in which cell behavior is dependent on the type of feature [20], [21], [22], [23], feature size and regularity [17] and cell type [18]. Examining the effect of pyramids of various heights on endothelial cell adhesion, our data may explain why different cell behaviors were observed in different reports.

The overriding conclusion from our study is that regardless of the surface chemistry, and indeed whether silicon was modified at all, surfaces that exhibit pyramidal features had considerably fewer adherent cells than flat surfaces. On non-flat surfaces, the degree of overall surface roughness i.e. the sizes of the pyramids hardly influenced the number of adherent cells. This result is consistent with previous studies that reported that substrates that do not display any particular orientation of topography negatively affected cell adhesion [30], [44].

This observation contrasts with our data on cell spreading and focal adhesion length. Even though the topography affected these parameters to some extent, the most striking observation was the following. Within a given topography, and this is true for all topographies assessed, cell spreading and focal adhesion length was determined by the surface chemistry and was greatest on surfaces with 6×10^8^ RGD/mm^2^ irrespectively of presence of pyramids and their size. This optimal density for cell spreading, which corresponds to an RGD-to-RGD spacing of 44 nm, fits well with those found on flat surfaces [10], [11], [12], [13], [14], [45], [46], [47].

Based on these two observations, *i.e.* that rougher surfaces facilitate less cell adhesion and that RGD density determines cell spreading, we propose that cell adhesion may be a two-step process. In the initial contact between cells and the substratum, the topography plays the dominant role and appears to override the effect of the surface chemistry. After initial contact, however, the surface chemistry of ligand presentation is the determining factor with regards to strengthening adhesion, cell spreading and focal adhesion formation. This model is consistent with a previous report suggesting that cells ‘tiptoe’ randomly across a surface before adherent to it [48]. Initial sampling of the surface architecture would also explain why flat and rough surfaces, or substrata with different types of features, evoke different levels of cell adhesion.

On flat surfaces and surfaces with nano-scaled roughness, we measured an optimal RGD density for cell adhesion of 6×10^5^ RGD/mm^2^, whereas a lower RGD density (below 6×10^5^ RGD/mm^2^) was optimal for adhesion on surfaces with micro-scaled pyramids. The biphasic response of cell adhesion can be explained if steric crowding of peptide ligands on the surface is taken into account. On closely packed surfaces (100% RGD coverage or 6×10^11^ RGD/mm^2^) it is likely that steric crowding of the peptides limits accessibility of the ligand to the cell surface receptors. This would affect the initial sampling of the surface, and hence the number of adherent cells, as well as cell spreading after the initial contact was made. Steric crowding of the peptide is less likely to occur on rough surfaces. In agreement with this statement is that the biphasic trend of cell adhesion was less pronounced on rough surfaces.

It appears that surfaces with micron-scaled pyramids modify cell behavior beyond the characteristics discussed so far. At higher magnification, actin and paxillin structures were visible in cells adherent to, or around, pyramids. Currently, the precise nature of these aggregates is unknown. Others have shown that on grooved substrata, focal adhesion proteins and actin filaments aligned along the grooves [49], [50]. Since only surfaces with micro-scaled pyramids hindered cell migration, it is also possible that the feature represents a physical constraint or barrier. This phenomenon was previously observed on substrates presenting V-shaped grooves, where the migration velocity of cells migrating across the grooves was significantly lower than the migration velocity of cells migrating in the direction parallel to the grooves [51].

Our data also revealed that RGD-to-RGD spacings of ∼44 nm on average is optimal for cell spreading. Spacings of 50–100 nm are physiologically relevant as collagen fibers, the main constituent of the extracellular matrix, have a periodic structure of ∼69 nm [52]. In fact, endothelial cells are able to re-organize layers of fibronectin fibers on polymer films in ubiquitous repeating units of ∼71 nm [53]. Given that the surface chemistry is a governing parameter in cell adhesion, our work also validates previous findings on flat surface regarding the effect of ligand presentation on focal adhesion structure [10], [11], [12], [13]. This is an important results as it shows that despite many previous studies where the topography was not controlled, for a given topography, once the cells adhere to the surface and form focal adhesions, cell behavior is largely dependent on the surface chemistry and density of adhesion points per surface area. This work confirms the complex interplay of topography and surface chemistry on endothelial cell adhesion and thus underlines the need for better control over these parameters in the design of biomaterials and implantable devices.

## Supporting Information

Figure S1 **AFM images of silicon (100) after different etching times.** (A) Silicon after 10 min and (B) 30 min etching. The presence of pyramid-like structures has been confirmed for etching times as short as 10 min. The sides of the features on surfaces etched for 10 min have a 64.2±7.2° angle with the base plane. Furthermore, these features have a shape which is not clearly defined as for surfaces etched for 30 min which present square based pyramids with side at a 56.3±2.7° angle with the base plane. This discrepancy in angle and shape for surfaces etched 10 min is due to an incomplete removal of all the facets other than the (111) face from the side of the feature.
(TIF)

Figure S2 **Cell spreading of adherent endothelial cells on silicon surfaces with different etching times.** Overview (A) and high magnification images of individual cells (B) incubated for 30 min with unmodified silicon with different surface topographies as determined by the etching times. Topographical characteristics are listed in Fig. 2. Cells were stained with phalloidin-Alexa Fluor 555 to visualize F-actin. The images show that cells spread and displayed an organized actin cytoskeleton, thus confirming cell adhesion. Scale bar in A is 40 µm, in B 10 µm.
(TIF)

Figure S3 **Surface chemistry without RGD ligand prevents endothelial cell adhesion and spreading.** Serum-starved endothelial cells were incubated for 30 min (A) or 3 h (B) with flat silicon surfaces modified with 100% EO_6_-OMe containing no RGD ligands or 1∶10^3^ EO_6_-RGD for comparison. In (A), cells were stained with phalloidin Alexa Fluor 555. In (B), endothelial cells expressing the focal adhesion protein Paxillin-GFP (green) were stained for F-actin with phalloidin Alexa Fluor 555 (red) and nuclei with DAPI (blue). Scale bar is 80 µm in A and 5 µm in B.
(TIF)

Figure S4 **Quantification of adherent endothelial cells on flat Si[111] and flat Si[100].** Flat Si[111] and Si[100] were functionalized with 100% EO_6_-RGD and incubated with serum-starved cells for 30 min. The number of adherent cells is similar for both surface types with no significant differences (p<0.05) confirming that crystal orientation has no discernable influence on cell adhesion.
(TIF)

Figure S5 **Quantification of focal adhesion length in adherent endothelial cells on flat and etched silicon surfaces.** Focal adhesion length in adherent endothelial cells after 3 h incubation with flat surfaces (A) and surfaces etched for 10 min (B), 30 min (C) and 90 min (D) for different RGD∶OMe ratios. Note that focal adhesions formed on pyramids will appear shorter as the pyramid angle is not taken into account. Data are derived from at least three independent surface preparations and 5 images per surface. Only focal adhesions located at the cell periphery were measured, the number of measured FA per image was >10. Error bars represent standard deviations.
(TIF)
