# Supplementary figures and images for: The Relative Importance of Topography and RGD Ligand Density for Endothelial Cell Adhesion
Source: PLoS One. 2011 Jul 11;6(7):e21869. doi: 10.1371/journal.pone.0021869 (PMC3136933; doi:10.1371/journal.pone.0021869)

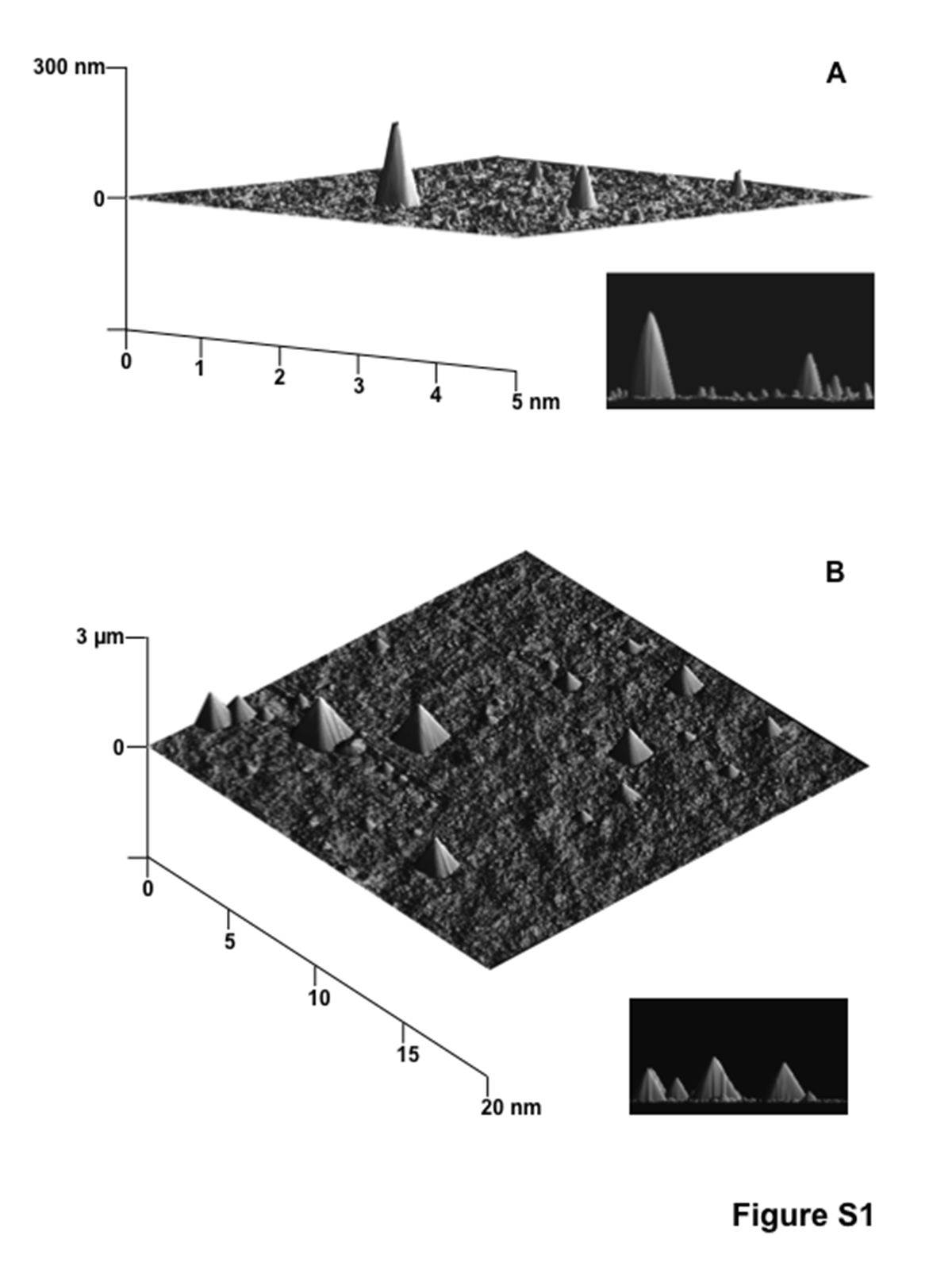

Supplement: Figure S1 — AFM images of silicon (100) after different etching times. (A) Silicon after 10 min and (B) 30 min etching. The presence of pyramid-like structures has been confirmed for etching times as short as 10 min. The sides of the features on surfaces etched for 10 min have a 64.2±7.2° angle with the base plane. Furthermore, these features have a shape which is not clearly defined as for surfaces etched for 30 min which present square based pyramids with side at a 56.3±2.7° angle with the base plane. This discrepancy in angle and shape for surfaces etched 10 min is due to an incomplete removal of all the facets other than the (111) face from the side of the feature. (TIF) [file pone.0021869.s001.tif]

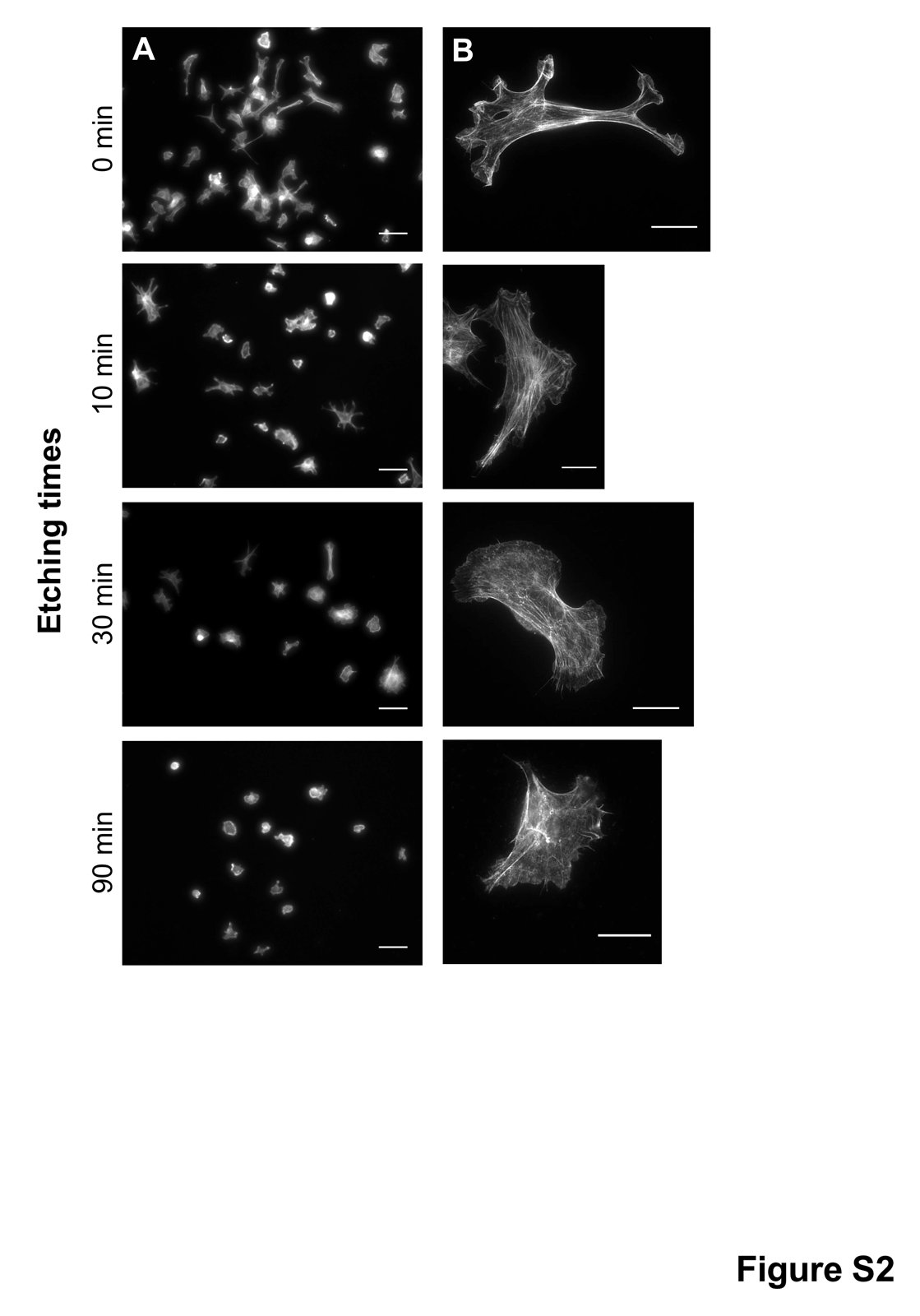

Supplement: Figure S2 — Cell spreading of adherent endothelial cells on silicon surfaces with different etching times. Overview (A) and high magnification images of individual cells (B) incubated for 30 min with unmodified silicon with different surface topographies as determined by the etching times. Topographical characteristics are listed in Fig. 2. Cells were stained with phalloidin-Alexa Fluor 555 to visualize F-actin. The images show that cells spread and displayed an organized actin cytoskeleton, thus confirming cell adhesion. Scale bar in A is 40 µm, in B 10 µm. (TIF) [file pone.0021869.s002.tif]

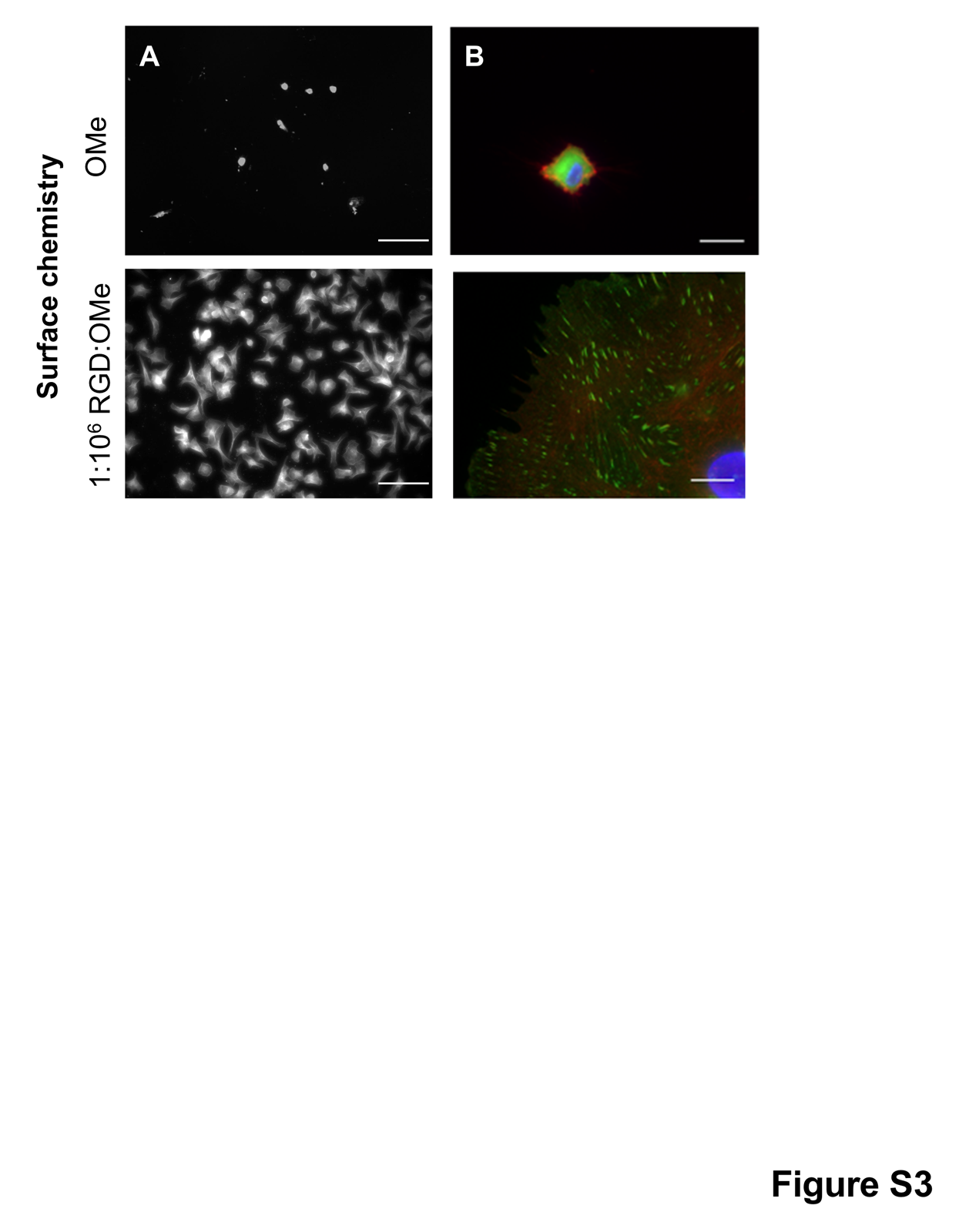

Supplement: Figure S3 — Surface chemistry without RGD ligand prevents endothelial cell adhesion and spreading. Serum-starved endothelial cells were incubated for 30 min (A) or 3 h (B) with flat silicon surfaces modified with 100% EO6-OMe containing no RGD ligands or 1∶103 EO6-RGD for comparison. In (A), cells were stained with phalloidin Alexa Fluor 555. In (B), endothelial cells expressing the focal adhesion protein Paxillin-GFP (green) were stained for F-actin with phalloidin Alexa Fluor 555 (red) and nuclei with DAPI (blue). Scale bar is 80 µm in A and 5 µm in B. (TIF) [file pone.0021869.s003.tif]

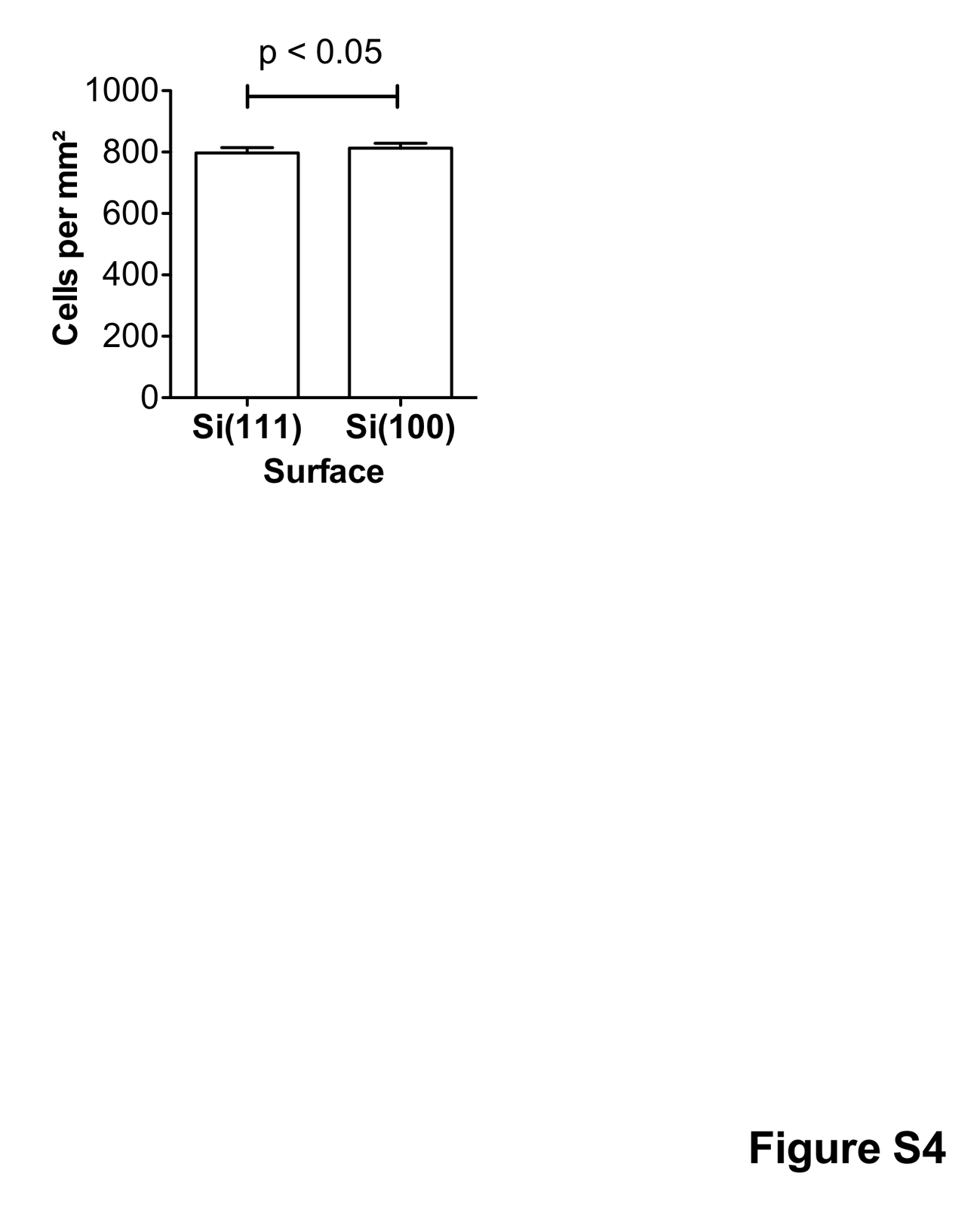

Supplement: Figure S4 — Quantification of adherent endothelial cells on flat Si[111] and flat Si[100]. Flat Si[111] and Si[100] were functionalized with 100% EO6-RGD and incubated with serum-starved cells for 30 min. The number of adherent cells is similar for both surface types with no significant differences (p<0.05) confirming that crystal orientation has no discernable influence on cell adhesion. (TIF) [file pone.0021869.s004.tif]

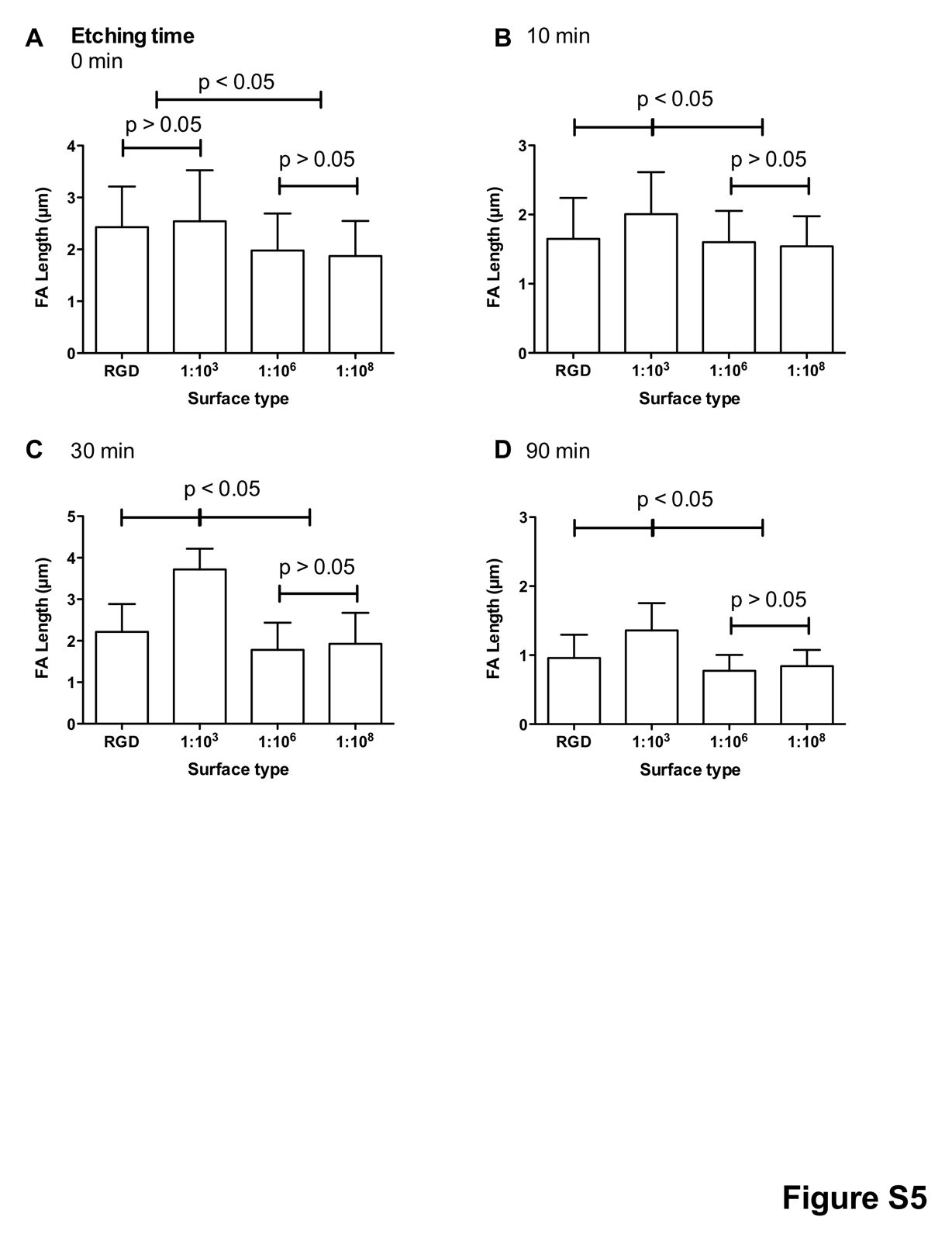

Supplement: Figure S5 — Quantification of focal adhesion length in adherent endothelial cells on flat and etched silicon surfaces. Focal adhesion length in adherent endothelial cells after 3 h incubation with flat surfaces (A) and surfaces etched for 10 min (B), 30 min (C) and 90 min (D) for different RGD∶OMe ratios. Note that focal adhesions formed on pyramids will appear shorter as the pyramid angle is not taken into account. Data are derived from at least three independent surface preparations and 5 images per surface. Only focal adhesions located at the cell periphery were measured, the number of measured FA per image was >10. Error bars represent standard deviations. (TIF) [file pone.0021869.s005.tif]
